# The effect of atmospheric doping on pressure-dependent Raman scattering in supported graphene

**DOI:** 10.3762/bjnano.9.65

**Published:** 2018-02-22

**Authors:** Egor A Kolesov, Mikhail S Tivanov, Olga V Korolik, Olesya O Kapitanova, Xiao Fu, Hak Dong Cho, Tae Won Kang, Gennady N Panin

**Affiliations:** 1 Belarusian State University, 4 Nezavisimosti Av., 220030 Minsk, Belarus; 2 Department of Chemistry, Moscow State University, Leninskie Gory, 1, b.3, 119991, Moscow, Russia; 3 Department of Physics, Quantum-Functional Semiconductor Research Center, Nano Information Technology Academy, Dongguk University, 3-26 Pildong, Junggu, 100-715, Seoul, Korea; 4 Institute for Microelectronics Technology & High Purity Materials, RAS, 142432 Chernogolovka, Moscow district, Russia

**Keywords:** adsorption, doping, graphene, pressure, Raman spectroscopy, substrate

## Abstract

Atmospheric doping of supported graphene was investigated by Raman scattering under different pressures. Various Raman spectra parameters were found to depend on the pressure and the substrate material. The results are interpreted in terms of atmospheric adsorption leading to a change in graphene charge carrier density and the effect of the substrate on the electronic and phonon properties of graphene. It was found that adsorption of molecules from the atmosphere onto graphene doped with nitrogen (electron doping) compensates for the electron charge. Furthermore, the atmosphere-induced doping drastically decreases the spatial heterogeneity of charge carriers in graphene doped with nitrogen, while the opposite effect was observed for undoped samples. The results of this study should be taken into account for the development of sensors and nanoelectronic devices based on graphene.

## Introduction

Graphene is a promising material for a variety of applications due to its unique physical properties [1]. Among its other outstanding features, one can distinguish its strong sensitivity to adsorbates, leading to a huge number of potential graphene applications in biosensors [2]. Atmospheric adsorption is known to affect graphene charge carrier density, leading to gradual self-sustained hole doping [3]. On one hand, adsorption can thus be an extremely undesirable effect when a nanoelectronic device is designed to operate on the basis of undoped properties of graphene; on the other hand, the adsorption of certain functional groups, for example, can be used as a constituent part of the methods of graphene functionalization [4]. At the same time, graphene layers on substrates are necessary for nanoelectronics. The substrates may affect the adsorption of graphene, either enhancing it, an effect very useful for biosensor applications [2], or reducing it, which is desirable when undoped graphene is needed as a device functional element.

A natural way for probing the adsorption properties of graphene is to conduct experiments under a low-pressure environment. Various high-pressure measurements for graphene are presented in the literature [5–6], while to the best of our knowledge, the studies of graphene’s response to pressure variations below atmospheric values are absent to date. As the pressure decreases, both graphene and the substrate will expand, and thus, the expected changes in the properties of graphene will not be only related to graphene’s compressibility, but also to strain caused by different compressibility values for graphene and the substrate. The authors of [5] have provided a convenient expression for compressibility-induced changes, while strain-related changes can be treated in a similar way to the thermal expansion description for supported graphene [7–8], where the strain is caused by a difference of thermal expansion coefficients for graphene and the substrate. Besides, monomers and functional groups adsorbed from the atmosphere are expected to become at least partially removed from the graphene surface as the pressure decreases, leading to changes in atmosphere-induced doping of the material [3]. A study of the characteristics of these processes may provide useful information about the adsorption properties of supported graphene.

Raman spectroscopy is a versatile tool for studying graphene and graphene-based nanomaterials [9–12]. Several different parameters of graphene’s Raman spectra are affected by charge carrier density variations, such as the G and 2D peak positions and the ratio of the 2D and G peak maximum intensities [13]. Since the sensitivity of these parameters to doping takes place due to fundamentally different processes, using a set of these parameters provides the possibility to accurately determine charge carrier density variations and distinguish the latter against, for example, substrate-induced strain effects [14–15].

The purpose of this study is to investigate the pressure-dependent behavior of the density of charge carriers in graphene and the effect of the substrate on the adsorption properties of graphene at pressures below atmospheric pressure.

## Results and Discussion

### Pressure-dependent Raman measurements

**Figure 1:**
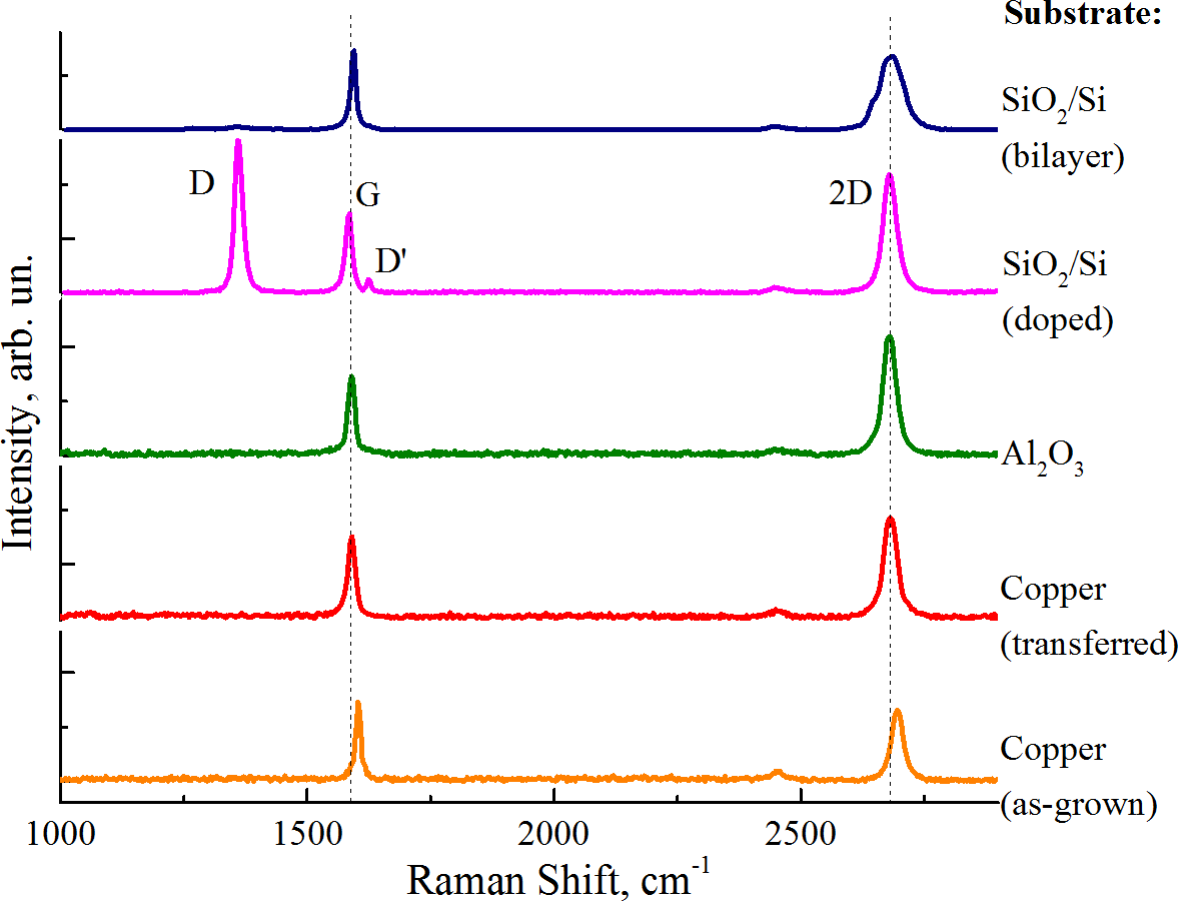
Raman spectra of graphene on various substrates, as well as bilayer and nitrogen-doped graphene.

Raman spectra of graphene on various substrates under atmospheric pressure are presented in Figure 1. Monolayer and bilayer graphene were identified by 2D peak approximations with one and four Lorentz functions, respectively, together with the 2D peak full-width at half-maximums (FWHMs). The *I*_2D_/*I*_G_ intensity ratio shows values of up to 1.5 for the monolayer and about 0.9 for bilayer graphene (the low *I*_2D_/*I*_G_ values can be explained by adsorption doping [9] and electrostatic doping effects [16], as analyzed throughout the article).

Comparing the spectra for as-grown and transferred graphene on copper, one can distinguish the upshifted positions of the G and 2D peaks. The stronger upshift for as-grown graphene (by 20–23 cm^−1^ with respect to normal positions) can be attributed to several effects: substrate-induced strain [14–15], hole doping by adsorbates [13], and substrate-induced electrostatic doping [16]. The G and 2D peak positions upshifted by 5–8 cm^−1^ and the considerably low *I*_2D_/*I*_G_ ratio of 1.3–1.4 for graphene transferred onto copper and Al_2_O_3_ could be explained by similar effects, which in this case, tend to be less pronounced after the transfer. For doped graphene (1.11% of nitrogen from XPS data), the D band intensity increases compared to pristine graphene, the second disorder-induced peak (the D’ band) also becomes evident, the G peak position is upshifted by ≈3 cm^−1^, while the 2D peak is about normal, naturally indicating electron doping together with *I*_2D_/*I*_G_ values of 1.4–1.6 [13].

**Figure 2:**
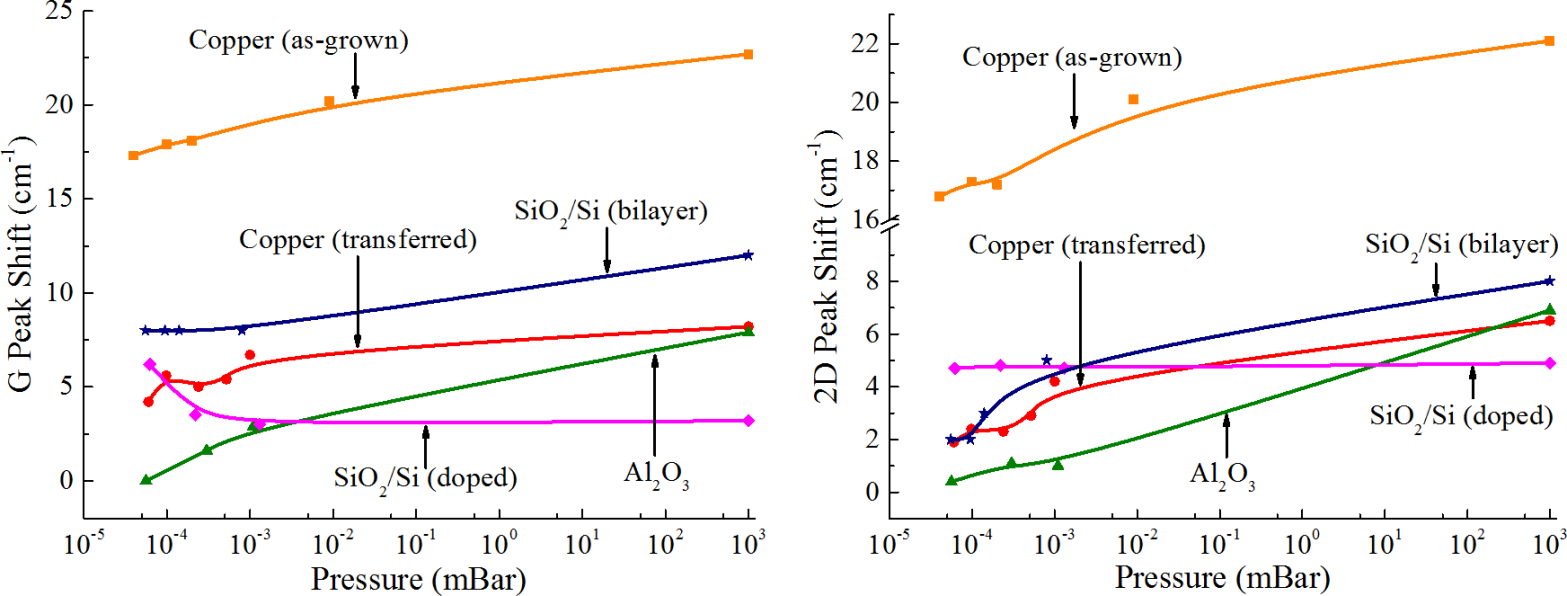
Pressure-dependent G and 2D peak position shifts for graphene on various substrates, as well as bilayer and nitrogen-doped graphene. Symbols show the experimental points, and solid lines represent general trends of peak shift.

Figure 2 shows pressure-dependent G and 2D peak shifts, typical for different samples, with respect to normal positions of about 1582 and 2676 cm^−1^ for a 532 nm laser, respectively. Downshift was commonly observed for undoped graphene peaks as the pressure decreased, while for nitrogen-doped graphene, the G peak position value increased. The 2D peak remained unchanged throughout the whole pressure range. Besides, changes of the *I*_2D_/*I*_G_ intensity ratio were observed as the pressure decreased, indicating variations of graphene charge carrier density [13]. After the atmosphere was returned to that of the pressure-controlled box, the spectra parameters gradually changed back to their initial values over a period of up to four days of air exposure.

### Theoretical estimation of different process contributions to the observed shift

The G and 2D peak experimental pressure shift 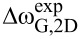 can be explained by changes in the corresponding phonon mode energy due to (1) graphene lattice compressibility 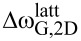 [5], (2) strain induced by the difference of compressibility values for graphene and substrate 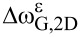 [5,14], and (3) possible desorption of atmosphere-induced doping groups 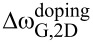 [3,13]. Thus, the experimental peak shift due to pressure change can be written as:

[1]
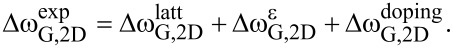


The first term is defined by the following expression [5]:

[2]
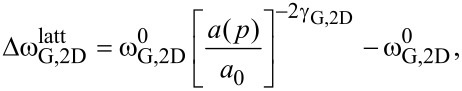


where 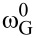 and 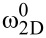 are normal G and 2D peak positions, respectively; γ_G,2D_ is a Grüneisen parameter for corresponding E_2g_ and A_1g_ phonon modes (values of 1.8 and 2.7 were used, respectively [14]), *a* is the graphene atomic bond length (*a*_0_ under zero-pressure conditions). As it was shown in [5], a relationship for graphite in-plane compressibility from [17] is applicable for *a*(*p*)/*a*_0_ estimation:

[3]
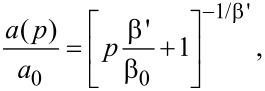


where


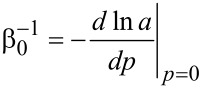


is the linear compressibility and β' is its pressure derivative. In the present study, β_0_ and β' values of 1250 GPa and 1 were used, respectively [17].

The strain-induced shift, by analogy with thermal expansion strain [7–8,18], can be written as:

[4]
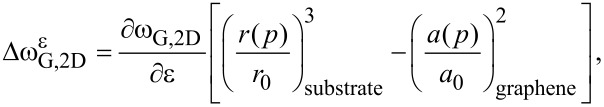


where


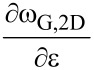


is a biaxial strain rate (in our calculations we used the values of −58 and −144 cm^−1^/% for G and 2D peak, respectively [14]), ε is a relative deformation (%), and *r* is a substrate atomic bond length.

In [5] it was shown that at low pressure the *r*(*p*)/*r*_0_ relation can be approximately defined through the substrate bulk modulus *B*_0_ in the following way:

[5]
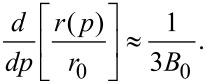


A value for the doping contribution 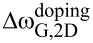 can on one hand be calculated from Equation 1 using the obtained experimental shift values; on the other hand, in can be obtained from other doping-dependent Raman spectra parameters that are not commonly referred to as strain-dependent, such as *I*_2D_/*I*_G_ intensity ratio [13].

During the calculation, it turned out that the graphene lattice compression contribution 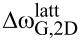 does not lead to a substantial change in the G and 2D peak positions – less than 10^−2^ cm^−1^ as the pressure changes from 0 to 1 atm (≈1·10^5^ Pa). The same is valid for the strain-induced shift 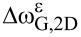 (less than 10^−3^ cm^−1^ for graphene on all substrates). Since these results dramatically exclude the first two terms from Equation 1, we assume the experimental shift to be caused by changes in graphene doping.

### Carrier density variation analysis

Table 1 demonstrates the carrier density change ∆*n* obtained from Raman spectra [13] of various graphene samples as the pressure decreased, as well as Raman feature parameters at normal and reduced pressures. As can be seen, greater ∆*n* values were obtained for nitrogen-doped graphene, while for bilayers on SiO_2_/Si and graphene transferred to copper, weaker desorption occurred. It is important to note that doping has various effects on the Raman spectral parameters [13]: stronger doping is expressed as lower *I*_2D_/*I*_G_ ratio values, while the G and 2D peak positions depend on carrier density non-linearly.

**Table 1:** Summarized Raman spectra parameters and resultant doping change as the pressure decreased from normal to reduced pressure for various graphene samples. For all samples except the nitrogen-doped graphene, ∆*n* indicates a hole density decrease (removal of hole-inducing adsorbates from the sample surface). For doped graphene, it corresponds to an increase of electron density (similar desorption process that cancel the balancing role of adsorbed groups).

Sample	Pressure, mbar	Pos(G), cm^−1^	Pos(2D), cm^−1^	*I*_2D_/*I*_G_	∆*n,* ×10^13^ cm^−2^

graphene on copper (as-grown)	1000	1605	2696	0.9	≈0.6–0.9 (hole doping)
	5 × 10^−5^	1599	2691	1.2	

graphene on copper (transferred)	1000	1590	2681	1.3	≈0.3–0.5 (hole doping)
	5 × 10^−5^	1586	2676	1.6	

graphene on Al_2_O_3_	1000	1590	2681	1.4	≈0.5–0.8 (hole doping)
	5 × 10^−5^	1582^a^	2674^a^	2.3^a^	

doped graphene on SiO_2_/Si	1000	1585	2679	1.5	≈0.7–1.0 (electron doping)
	5 × 10^−5^	1588	2679	1.2	

bilayer on SiO_2_/Si	1000	1594	2682	0.9	≈0.3–0.5 (hole doping)
	5 × 10^−5^	1590	2676	1.0	

^a^The closest to normal graphene peak positions as well as greater *I*_2D_/*I*_G_ values were observed for graphene on Al_2_O_3_ at reduced pressure.

In order to analyze the obtained results, we first compare the peak position shift for pristine and transferred graphene on Cu in Figure 2, where one can notice a similar behavior up to a certain constant. A considerable similarity of the peak position downshift indicates analogous kinetics of adsorbate removal from graphene surface. However, a different intercept leads to a 13–15 cm^−1^ distance between the plots. This observation can be explained by substrate-induced strain in as-grown graphene (leading to greater overall peak positions [14]): The van der Waals graphene–substrate interaction model may not be fully applicable for this case due to the possibility of chemical bonds present between graphene and copper [19] which are terminated after the transfer. Besides, this may lead to different values of the average graphene–substrate distance resulting in deviation of the density of states (DOS) from a simple Dirac cone [16] and a consequent shift of the Dirac point energy, leading to different substrate-induced doping that affects all Raman features related to carrier density but most likely do not change with the pressure (smaller overall values of *I*_2D_/*I*_G_ intensity ratio). This explains the similar shift but different intercept for pristine and transferred graphene on Cu in Figure 2.

Comparing graphene transferred to Cu and Al_2_O_3_ as typical conducting and dielectric materials, a stronger carrier density change was obtained for the latter. As the pressure decreases, removal of adsorbates from the surface of graphene on Al_2_O_3_ results in the Raman spectra parameters becoming close to those typical for completely undoped graphene [13] – in particular, the strong *I*_2D_/*I*_G_ increase seen in Table 1 should be noted. At the same time, a smaller hole density decrease for graphene transferred to Cu can be explained by analogy with as-grown graphene by stronger graphene–substrate interaction in this case (interaction energy of 0.72 J/m^2^ against ≈0.47 J/m^2^ for graphene on Al_2_O_3_ substrate [20–21]) and therefore more pronounced substrate-induced doping [16]. Besides, the electrostatic screening of the electron–electron interaction for graphene on a metallic substrate [22–23] can contribute. The screening effect results in renormalization of the Fermi velocity, Dirac point velocity, and overall distortion of the Dirac cone [24], leading to a possible charge carrier density increase [25].

In the case of bilayer graphene, the desorption process leads to a density decrease of about 0.4 × 10^13^ cm^−2^ hole, which is close to the case of graphene transferred to Cu. Being a van der Waals structure on the scale of adsorption effects (in terms of interlayer bonding), bilayer graphene is known to have a slightly greater energy barrier for the adsorption process [26]. However, adsorption in this case can also lead to at least partial layer decoupling that results in the presence of biaxial strain [27]. Besides, values of *I*_2D_/*I*_G_ did not indicate a strong doping presence. Thus, the overall greater G and 2D peak position values most likely are strain-related rather than an indication of greater atmosphere-induced doping.

A very interesting situation is observed for nitrogen-doped graphene. The results of pressure-dependent studies presented in Figure 2 have shown a G peak upshift, while the 2D peak position remained unchanged. This behavior together with the change of *I*_2D_/*I*_G_ corresponds to an increase of electron density, according to dependencies presented in [13]. This fact leads to an interesting conclusion that adsorption hole doping most likely counterbalances electron concentration in nitrogen-doped graphene. A greater change of the carrier density within the experimental pressure range compared to undoped graphene indicates that under electronic doping more active adsorption occurs, balancing the “excess” electron density through opposite-sign charge carrier introduction and subsequent recombination, and moving the Fermi level position closer to a normal value.

**Figure 3:**
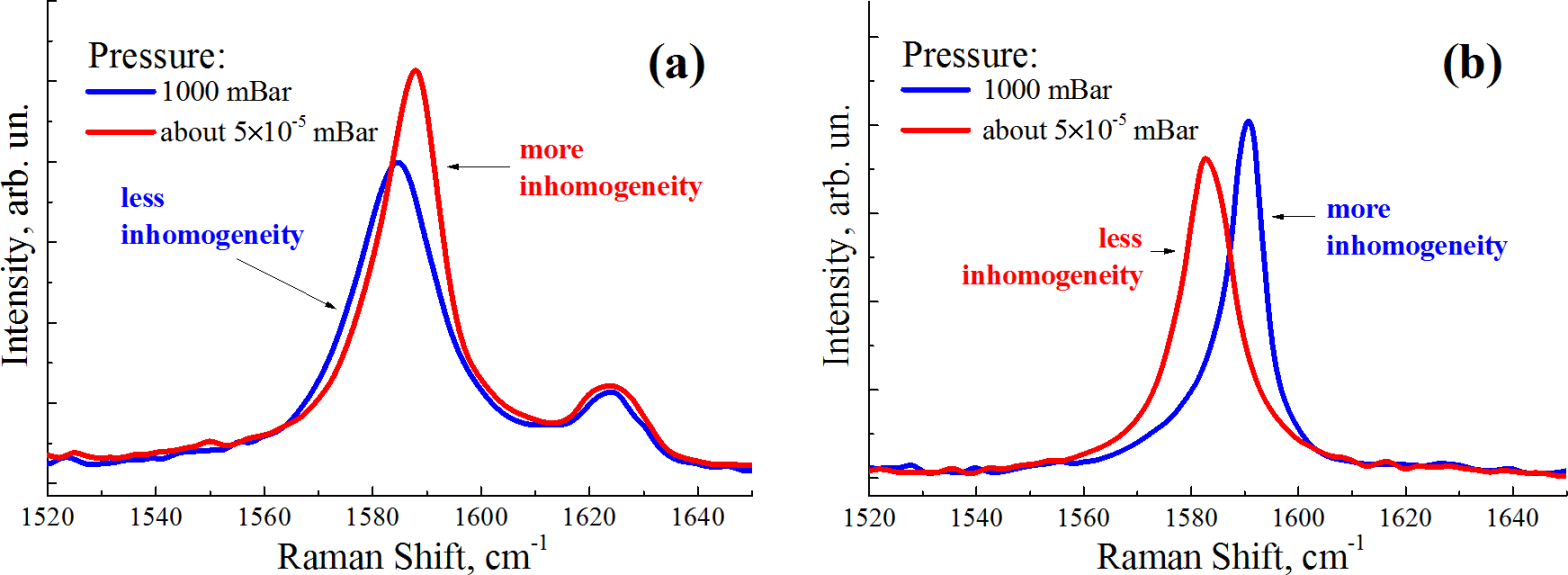
G peak profile for (a) nitrogen-doped graphene on SiO_2_/Si and (b) graphene on Al_2_O_3_ at normal and reduced pressures.

As seen from Figure 3a, in addition to an upshift, the initially distorted G peak at normal pressure experiences stronger profile distortion as the pressure decreases, with the most asymmetric profile corresponding to the lowest pressure. This fact indicates that as desorption occurs, inhomogeneity of the charge carrier distribution increases, approaching conditions typical for doped graphene [28]. Thus, atmospheric adsorption not only counterbalances the excess electron concentration, but also reduces the charge carrier inhomogeneity, with the whole system evolving closer to equilibrium.

It is important to note that the opposite situation was observed for undoped graphene: as the pressure decreased (with desorption leading to doping decrease), the G peak (in addition to the downshift) evolved from a distorted lineshape at normal pressure to an almost symmetric one in vacuum (≈5 × 10^−5^ mbar), as seen in Figure 3b. This fact means that the effect of atmospheric adsorption that reduces charge carrier inhomogeneity in graphene is quite unique for the electron-doped graphene case, while the inverse process is naturally typical for the pure material, as it was previously noted [28].

The results presented above demonstrate that pressure-dependent Raman spectroscopy is a convenient tool for probing atmosphere-induced doping in graphene. Low-pressure behavior of adsorbates present on the graphene surface and concomitant charge carrier properties strongly depend on the substrate material, as well as graphene doping and number of layers. These results can be taken into account during the development of any graphene-related devices, either specifically involving graphene adsorption properties (for example, biosensors) or assuming exposure of air to the device functional elements.

## Conclusion

Pressure-dependent Raman spectroscopy studies in the range from 1000 to 5 × 10^−5^ mbar of supported graphene have shown that the G and 2D peak positions and the ratio of the 2D and G peak intensities change with the pressure, whereby the substrate material affects the dependencies. Performed calculations showed negligible lattice compressibility and strain contributions to the observed effect, and thus the shift was fully attributed to atmosphere-induced adsorption doping. During the analysis, the effect of the substrate on graphene electronic and phonon properties was taken into account. For nitrogen-doped graphene (electronic doping), adsorption was found to counterbalance the excess charge carriers. In addition, it turned out that atmosphere-induced doping decreases charge carrier inhomogeneity in nitrogen-doped graphene. For the undoped graphene samples, the opposite effect occurs. The results of the present study are useful for graphene-based sensor design, graphene functionalization techniques, and taking into account adsorption effects during nanoelectronic device engineering.

## Experimental

Graphene was synthesized on Cu foil at 1020 °C by a chemical vapor deposition (CVD) method using a mixture of CH_4_ of 40 sccm and H_2_ of 10 sccm. Cu foil (Alfa Aesar, 99.999%, 10 × 30 cm^2^, 25 μm thick) was pre-annealed at 1060 °C with a hydrogen flow of 300 sccm and an argon flow of 2,000 sccm at a pressure of <10^−4^ Torr for 1–2 hours inside the chamber. After the growth of graphene, the Cu foil was cooled to room temperature. The cooling rate was described in detail elsewhere [29].

The grown graphene was transferred to various substrates using a “PMMA-mediated” method [30]. PMMA (molecular weight = 996 000, dissolved in anisole) was spin-coated (3000 rpm, 1 min) on graphene supported by Cu foil. A 0.1 M (NH_4_)_2_S_2_O_8_ aqueous solution was used to etch Cu and a water/isopropyl alcohol mixture was used to wash graphene from etching products [31]. PMMA was removed by submerging the sample in glacial acetic acid (extra pure) [32] for 4 h.

Graphene, doped with nitrogen, was prepared using a nitrogen-plasma treatment described elsewhere [33].

The Raman spectra were obtained with a confocal Raman spectrometer Nanofinder HE (LOTIS TII) with a spectral resolution better than 3 cm^−1^. For the excitation of Raman radiation, a continuous wave solid-state laser with a wavelength of 532 nm was used. During low-pressure Raman measurements, the sample was studied in a pressure-controlled box (pressure ranged from 1000 to about 5 × 10^−5^ mbar). A laser power of 2 mW was used, and the laser spot diameter was about 1.5 µm. Pressure-dependent measurements were carried out in a typical graphene monolayer and a bilayer low-defect region.
